# Editorial

**DOI:** 10.1007/s00103-022-03587-y

**Published:** 2022-09-29

**Authors:** Jürgen Bengel, Uwe Koch-Gromus

**Affiliations:** 1 Institut für Psychologie, Albert-Ludwigs-Universität Freiburg, Engelbergerstr. 41, 79085 Freiburg, Deutschland; 2 Institut und Poliklinik für Medizinische Psychologie, Universitätsklinikum Hamburg-Eppendorf, Martinistr. 52, 10246 Hamburg, Deutschland

Der Rettungsdienst sorgt für die präklinische Notfallversorgung von Menschen mit akuten Erkrankungen, Verletzungen und Vergiftungen, die nicht durch niedergelassene Ärzt:innen und ambulante Einrichtungen behandelt werden können. In der Versorgung der gesundheitlichen Notfälle wird unterschieden zwischen dem bodengebundenen Rettungsdienst (Notfallrettung, Krankentransport) und der Luftrettung sowie dem Bergrettungsdienst, der Höhlenrettung und der Wasserrettung. Der bodengebundene Rettungsdienst wird in Deutschland je nach Region durch Arbeiter-Samariter-Bund, Deutsches Rotes Kreuz, Johanniter-Unfall-Hilfe, Malteser-Hilfsdienst, Feuerwehr sowie kommunale oder private Rettungsdienstunternehmen organisiert.

Der Rettungsdienst kooperiert eng mit der Polizei, der Feuerwehr und anderen Hilfsorganisationen sowie den Notfallambulanzen und den Krankenhäusern. Er wird in der Regel über Rettungsleitstellen alarmiert und koordiniert und ist nicht zu verwechseln mit dem ärztlichen Notdienst. Das Rettungspersonal setzt sich in der Regel aus Notärzt:innen, Notfallsanitäter:innen und Rettungssanitäter:innen zusammen. Qualifizierte ehrenamtliche Notfallhelfer:innen (First Responder) ergänzen vielerorts das Personal. Der Rettungsdienst hat sich in den letzten Jahrzehnten immer mehr professionalisiert und dynamisch entwickelt. Integrierte Leitstellen und gut qualifiziertes Personal sind die Regel ebenso wie kurze Hilfsfristen im urbanen Bereich. Standardisierte Abfragen und aufgezeichnete Protokolle der Leitstellen garantieren eine Dokumentation der Meldung und des Einsatzes. Die Leitstellenarbeit, das Meldesystem und das Einsatzmonitoring basieren auf moderner Technologie. Auch die Ausstattung der Rettungswagen und die Transporttechniken sind in Deutschland auf gutem Niveau.

Die Organisation der Rettungsmittel, das Rettungsdienstpersonal und der notärztliche Dienst arbeiten meist im „Rendezvous-System“ – ein Verfahren, bei dem verschiedene Einheiten zum selben Einsatzort alarmiert werden. Eine möglichst kurze „Hilfsfrist“, also der Zeitraum zwischen der Meldung eines Notfalls und dem Eintreffen des Rettungsdienstes, ist insbesondere im ländlichen Raum eine große Herausforderung. Die Notfalltelemedizin, die sich in den letzten Jahren merklich weiterentwickelt, kann künftig die Ersteinschätzung und die Notfallversorgung insgesamt ergänzen und wichtige Impulse geben.

Die zweijährige Ausbildung zum Rettungsassistent:in galt bis 2014, seither ist die dreijährige Ausbildung als Notfallsanitäter:in Standard. Die Zusammenarbeit von Notfallsanitäter:innen und Notärzt:innen gelingt in der Regel sehr gut; Fragen der Delegation stellen sich jedoch immer wieder.

Besondere Konstellationen hinsichtlich spezifischer Kompetenz und Belastung für die Einsatzkräfte bilden u. a. psychiatrische Notfälle, Fälle im Zusammenhang mit Drogenkonsum und Kindernotfälle. Ein weiteres relevantes Thema ist der Eigenschutz des Rettungspersonals u. a. im Hinblick auf Gefahren, wie etwa den Kontakt mit Giftgas oder Krankheitserregern, gefährliche Einsatzsituationen, aber auch Aggression und körperliche Gewalt gegen Einsatzkräfte. Zuschauer bei Unfällen und Bergungen können das Personal zeitlich und psychisch belasten.

Die psychosoziale Versorgung des Rettungsdienstpersonals nach kritischen Einsätzen ist Thema und Auftrag der Rettungsdienstorganisationen und der ärztlichen Fachgesellschaften. Die notfallmedizinische Versorgung wurde auch für die Patient:innen um die psychosoziale Erstversorgung erweitert, u. a. durch Kriseninterventionsteams und Notfallseelsorge für Betroffene und Angehörige. Neben der professionellen Notfallversorgung ist die Erste Hilfe durch Laien, u. a. die Laienreanimation, hoch bedeutsam. Wissensvermittlung und Motivation zur Hilfe sind genauso wichtig wie das Bereitstellen von Defibrillatoren.

Dieses Themenheft des Bundesgesundheitsblattes greift in insgesamt 11 Beiträgen wichtige Aspekte der präklinischen Notfallversorgung auf: Die ersten beiden Beiträge befassen sich mit lebensrettendem Handeln am Notfallort.

*L. Horriar et al. *beschreiben die Wiederbelebung durch Laien als eine zentrale Maßnahme, um die Überlebenschancen nach Herz-Kreislauf-Stillstand zu erhöhen. Den vom European Resuscitation Council aktualisierten Reanimationsleitlinien 2021 mit ihrem Fokus auf Laienreanimation wird von den Autor:innen in diesem Kontext besondere Bedeutung zugeschrieben.

*B. Schmid et al. *betonen in ihrem Beitrag die Wichtigkeit der kompetenten präklinischen Ersteinschätzung durch den Rettungsdienst am Einsatzort für die weitere Versorgung der Notfallpatient:innen. Die präklinische Ersteinschätzung dient vor allem der adäquaten Notfallversorgung vor Ort, der Indikationsstellung für eine Einweisung und der Auswahl des weiterversorgenden Krankenhauses. Sie setzt eine hohe fachliche Kompetenz aller beteiligten Akteure voraus.

Die folgenden 3 Beiträge beziehen sich auf Managementaspekte der Notfalleinsätze.

*D. Lauer et al. *beschreiben die hohen Anforderungen, die an das aktuelle Rettungsdienstmanagement gestellt werden. Die Herausforderungen werden sich vor dem Hintergrund stark steigender Einsatzzahlen in den meisten Rettungsdienstbereichen und neuer technischer sowie medizinischer Aufgabenstellungen weiter erhöhen. Die Autor:innen formulieren eine Reihe von Anregungen für die Weiterentwicklung des Managements der Notfallversorgung.

*F. Dax et al.* berichten über Ergebnisse einer auf Bayern bezogenen Untersuchung zur Frage, welche Faktoren dafür bestimmend sind, ob Notfalleinsätze mit oder ohne Patiententransport erfolgen. Die Analyse der Einsatzgründe zeigt, dass die Inanspruchnahme des Rettungsdienstes durch die Alarmierungsplanung beeinflusst wird. Damit dürfte den Leitstellen in diesem Bereich ein erhebliches (bisher ungenutztes) Potenzial zur Steuerung und Verbesserung der Ressourcenallokation zukommen.

*P. Brinkrolf et al. *stellen Ergebnisse einer Studie vor, in der geprüft wird, ob ein Telenotarztsystem im ländlichen Raum eine effiziente Ergänzung der präklinischen Notfallversorgung darstellen kann. Die Autor:innen kommen auf der Basis einer umfangreichen Datengrundlage zu der Einschätzung, dass die Implementierung des Telenotarztsystems mit klinischen und ökonomischen Vorteilen verbunden ist.

Die nachfolgenden 5 Beiträge thematisieren sehr unterschiedliche psychosoziale Aspekte der Arbeit des Rettungswesens.

*S. Hoppe *beschreibt und analysiert zunächst die Arbeit der Psychosozialen Akuthilfen (PSAH) als Teilbereich der Psychosozialen Notfallversorgung (PSNV). Teams der Krisenintervention und Notfallseelsorge konzentrieren sich auf Angehörige und Hinterbliebene, Vermissende, Augenzeug:innen und Überlebende von kritischen Ereignissen und bieten unmittelbar ereignisbezogene psychosoziale Unterstützung an. Der Artikel gibt einen umfassenden Überblick über die Arbeit der PSAH und beschreibt deren Struktur, Handlungslogik und Ziele.

*S. Finkeldei et al. *thematisieren die Besonderheiten der Aufgabenstellungen der Psychosozialen Notfallversorgung (PSNV) bei der Zielgruppe Kinder. Gestützt auf Forschungsergebnisse der Psychotraumatologie und der Psychosozialen Notfallversorgung werden insbesondere die Auswirkungen des Bezugspersonenverhaltens auf die kindliche Verarbeitung bei Notfallereignissen fokussiert. Darauf aufbauend formulieren die Autor:innen Empfehlungen für die Akutbetreuung von Kindern und diskutieren die besonderen Herausforderungen dieses Praxisfeldes.

*I. Böckelmann et al. *untersuchen die psychischen und physischen Belastungen von Einsatzkräften des Rettungsdienstes und analysieren deren Zusammenhänge mit arbeitsbezogenem Verhalten und Beanspruchungsfolgen. Vor allem bei den Einsatzkräften mit einem Risikomuster des arbeitsbezogenen Verhaltens weisen die Analysen auf Ansatzpunkte für organisations- und individualbezogene präventive Maßnahmen hin.

*H. Karutz *setzt sich in seinem Beitrag mit dem zuschauenden Verhalten an Unglücksorten auseinander und hinterfragt bzw. relativiert die häufig in den Medien präsentierten Berichte über stark störendes Verhalten von „Gaffern“ bzw. „Schaulustigen“ bei Rettungsarbeiten an Unglücksorten. Sein Überblick zeigt, dass zuschauendes Verhalten an Unglücksorten keineswegs allein auf „Neugier“ und „Schaulust“ zurückzuführen ist, sondern wesentlich komplexere Erklärungsmuster verlangt.

*F. Leuschner et al. *befassen sich in ihrem Beitrag mit einem ebenfalls häufig und intensiv in den Medien diskutierten Thema, nämlich Gewalt gegen Rettungsdienstpersonal. Die Ergebnisse eines Forschungsvorhabens der Autor:innen zeigen, dass Angriffe, insbesondere verbaler Art, zum Arbeitsalltag von Rettungsdienstpersonal gehören. Sie plädieren für eine Verbesserung der Ausbildung und Weiterbildung des Personals, für Deeskalationsstrategien und für Maßnahmen der Eigensicherung, um das Risiko von Angriffen und Belastungen im Berufsalltag zu senken.

Der letzte Beitrag des Themenheftes befasst sich mit den Bildungsperspektiven der Notfallsanitäter:innen. *P. Dahlmann et al. *setzen sich mit der Frage auseinander, wie die Ausbildung des Personals bei den sich aktuell stark verändernden gesellschaftlichen Anforderungen an das System der Notfallversorgung und des Rettungsdienstes angepasst werden sollte. Dabei schreiben sie der Weiterentwicklung der Ausbildung der Notfallsanitäter:innen im Sinne von evidenzbasierter und patientenzentrierter Versorgung eine zentrale Bedeutung zu.

Wir hoffen, liebe Leser:innen, dass wir Ihnen mit dem Themenheft ein breites und interessantes Spektrum zur aktuellen Situation des Rettungsdienstes in Deutschland zusammengestellt haben.

Ergänzend findet sich in diesem Heft die Vorstellung eines Projektes aus dem Katastrophenschutz mit dem Titel „Lagebild Bevölkerungsverhalten für ein effektives staatliches Krisenmanagement (LB BevV)“. Dieses Projekt ist im Referat Psychosoziales Krisenmanagement im Bundesamt für Bevölkerungsschutz und Katastrophenhilfe (BBK) angesiedelt. In einem „Lagebild Bevölkerungsverhalten“ sollen zukünftig die aktuellen Bedarfe, Bedürfnisse und Ressourcen der Bevölkerung in einer Krise dargestellt werden, um das staatliche Krisenmanagement zu unterstützen und Maßnahmen gezielt anzupassen.

Wir wünschen Ihnen eine spannende Lektüre.

Jürgen Bengel und Uwe Koch-Gromus

